# Mechanical Properties and In Situ Deformation Imaging of Microlattices Manufactured by Laser Based Powder Bed Fusion

**DOI:** 10.3390/ma11091663

**Published:** 2018-09-09

**Authors:** Anton Du Plessis, Dean-Paul Kouprianoff, Ina Yadroitsava, Igor Yadroitsev

**Affiliations:** 1 CT Scanner Facility, Stellenbosch University, Stellenbosch 7602, South Africa; 2 Department of Mechanical Engineering, Central University of Technology, Free State, Bloemfontein 9300, South Africa; dkouprianoff@cut.ac.za (D.-P.K.); iyadroitsava@cut.ac.za (I.Y.); iyadroitsau@cut.ac.za (I.Y.)

**Keywords:** laser powder bed fusion, additive manufacturing, X-ray tomography, in-situ imaging, Ti6Al4V, lattice structures

## Abstract

This paper reports on the production and mechanical properties of Ti6Al4V microlattice structures with strut thickness nearing the single-track width of the laser-based powder bed fusion (LPBF) system used. Besides providing new information on the mechanical properties and manufacturability of such thin-strut lattices, this paper also reports on the in situ deformation imaging of microlattice structures with six unit cells in every direction. LPBF lattices are of interest for medical implants due to the possibility of creating structures with an elastic modulus close to that of the bones and small pore sizes that allow effective osseointegration. In this work, four different cubes were produced using laser powder bed fusion and subsequently analyzed using microCT, compression testing, and one selected lattice was subjected to in situ microCT imaging during compression. The in situ imaging was performed at four steps during yielding. The results indicate that mechanical performance (elastic modulus and strength) correlate well with actual density and that this performance is remarkably good despite the high roughness and irregularity of the struts at this scale. In situ yielding is visually illustrated.

## 1. Introduction

Additive manufacturing (AM) is an emerging production technique whereby a part with complex geometry can be produced directly from a design file in a layer-by-layer method [1,2]. In the case of laser-based powder bed fusion (LPBF), a single layer of the part is selectively fused using a laser beam that is scanned across a powder bed surface in a series of tracks, new powder is delivered, and the next layer is scanned and fused. Predictably, the part integrity requires that single tracks are stable [3] and well overlapped with one another, as well as layers to prevent unwanted porosity in solid parts. This has been discussed in some detail in a recent review of the use of X-ray microtomography in additive manufacturing [4]. Despite the possibility of irregularities in parts, it is possible to produce parts with excellent mechanical properties when process parameters are optimized (see, for example, Reference [5] for biomedical Ti6Al4V produced by LPBF).

One of the major benefits brought about by additive manufacturing is the ability to produce complex parts, and this is especially true for lattice structures that are regularly spaced and repeating combinations of struts with spaces between them. Lattice structures produced by AM have been the topic of many studies in recent years due to the potential to use these in bone replacement implants [6,7,8]. In implants, the porous nature of the lattice structure is beneficial to lower the elastic modulus of biocompatible materials to match that of the bone at the implant interface, minimizing the possibility for stress shielding causing loss in bone density in the vicinity. Additionally, the open porous nature allows for bone ingrowth into the lattice, effectively ensuring a good bond with the existing bone.

The investigation of the mechanical properties of lattices produced by AM, and in particular LPBF, is therefore crucial for the adoption of this type of design in implants, along with tailoring its properties for the application of custom shapes that meet local bone density requirements. In general, the mechanical properties of these structures can be predicted by the Ashby–Gibson model for open-cell foams [9,10], with a general relationship for elastic modulus of the lattice (*E*) as a function of the lattice density (*ρ*) and elastic modulus of the solid material used (*E_s_*), given as follows:

(1) $$E=\propto_{2}E_{s}{\left(\frac{\rho }{\rho_{S}}\right)}^{2},$$

where ∝_2_ is a value between 0.1 and 4 depending on the lattice geometry [9].

In early work by Parthasarathy et al. [11], simple cubic lattices of Ti6Al4V produced by electron beam melting were analyzed by microCT and mechanical testing and it was found that the mechanical properties are weaker than predicted and this was especially so for a model with thinner struts. This might be attributed to manufacturing irregularities such as the rough as-built surface and unexpected porosity inside the struts. Geometric accuracy is often a limitation in additive manufacturing of cellular structures, as is the entrapment of powder in the small pore spaces of these structures [1]. Various LPBF cellular structures in Ti6Al4V have been produced in different unit cell designs and their mechanical properties investigated, for example cubic [12], diamond [13], and combinations of designs including body-centered cubic [14] and minimal surfaces [15]. Besides variations in mechanical performance induced by geometric inaccuracy and manufacturing errors, slight variations also exist in the properties of various lattice designs themselves. This was demonstrated recently by the numerical analysis of various lattice designs, ignoring manufacturing imperfections [16].

It is therefore clear that the only way to fully understand the complex behavior of lattice structures (with many variations in designs and varying amounts of manufacturing errors, which to some extent also depend on the design), is to use high resolution imaging. In prior work, using relatively large lattices with struts more than 1 mm in diameter, compression tests combined with microCT imaging was used to visualize the first yielding crack locations, as shown in Reference [17], with loads up to 140 kN. This was done ex situ by stopping the mechanical test at first yielding and correlating “before” and “after” microCT scans to find cracks/yielding locations. Some work has also previously been done using in situ synchrotron tomography during the loading of small unit cells produced by LPBF [18]. This work showed local strut-scale deformations during yielding and compared experimental results to those predicted by simulation, but was limited to unit cells, which are not necessarily representative of tessellated lattices. Furthermore, the effect of LPBF process parameters on the morphology and mechanical properties of small lattices were investigated using a combination of methods, including microCT, where it was shown that properties may be improved by process optimization and failure occurred at the nodes in that case [19].

In this work, the aim was to investigate the smallest possible lattices that can be produced with a typical LPBF system with a track width of roughly 0.1 mm. In addition to investigating the mechanical properties of such small lattices, the accuracy of these produced microlattices may be useful as a reference for future work. Four different sizes of lattice cube samples were produced, each containing six unit cells in each direction with a diamond unit cell design with porosity of 80% and unit cell sizes 0.6, 0.8, 1.0, and 1.2 mm. Since the geometry and the density is kept constant, the theoretical elastic modulus and yield stress should be identical in all four cases, therefore the aim was to investigate the properties as the struts become thinner with decreasing unit cell size. The mechanical properties of these small lattices is reported and in situ imaging of the lattice deformation using high resolution X-ray tomography is demonstrated.

## 2. Materials and Methods

Models were designed in Materialize Magics [20] and produced from Ti6Al4V extra low interstitials (ELI) powder by EOSINT M 280 (EOS GmbH—Electro Optical Systems, Krailling, Germany) with a 200 W laser and original parameters Ti64_Performance 1.1.0 (30 μm). Gas atomized Ti6Al4V ELI powder from TLS Technik GmbH & Co. Spezialpulver KG (Bitterfeld-Wolfen, Germany) was used. Particle size distribution was as follows: equivalent diameters (weighted by volume) *d*_10_ = 12.1 µm, *d*_50_ = 23.6 µm, and *d_9_*_0_ = 37.6 µm. The chemical composition fulfilled the requirements of ASTM F136 standard specification for wrought Ti6Al4V ELI alloy for surgical implant applications regarding maximum concentration of impurities (ASTM International, West Conshohocken, PA, USA).

**Figure 1:**
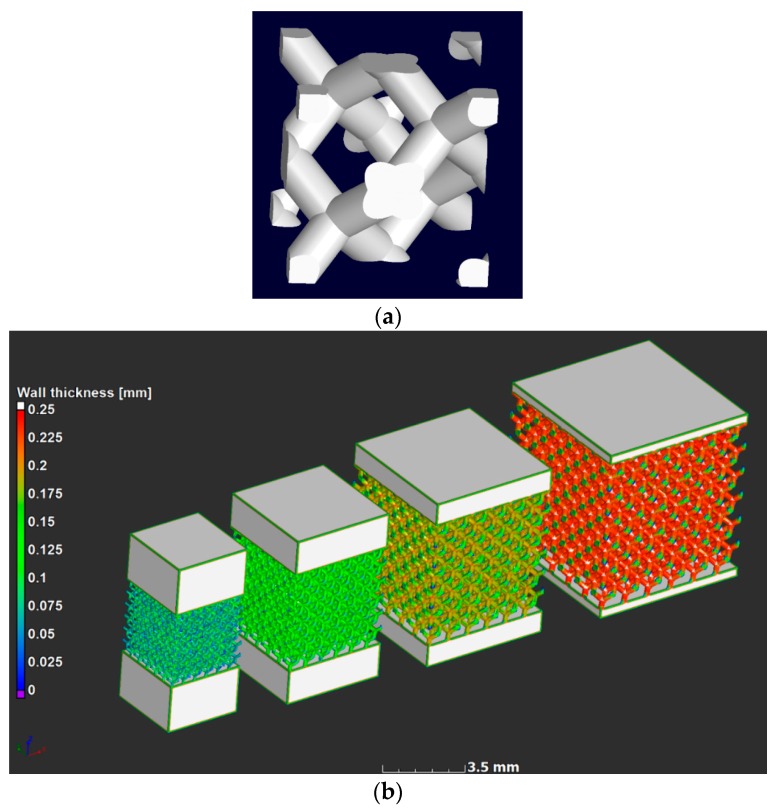
Design of microlattices showing (**a**) a single unit cell design of diamond type, and (**b**) the four full CAD lattice design used with unit cell sizes 0.6, 0.8, 1.0 and 1.2 mm, with strut thickness analysis.

A stress-relief cycle for 3 h at 650 ℃ [5] was conducted in an argon atmosphere after producing the parts, after which the parts were cut from the build plate using electrical discharge machining. The unit cell design used in this work was the diamond design; the unit cell is shown in Figure 1a. Three samples of each of four designs were produced, the computer aided design (CAD) designs are shown in Figure 1b, with a strut thickness analysis showing that the larger the unit cell, the thicker the strut was, as expected. Strut thickness analysis allowed measurement of the “wall” thickness at every point in the structure. In this case the sphere method was used, which provided the value of the maximal-fitted sphere in every point in the structure. The designs were selected to produce cubes with six unit cells in each direction, with unit cell sizes for the four designs being 0.6, 0.8, 1.0, and 1.2 mm. This ensured that the density was kept constant and was selected to be 20% dense (80% porosity). The physical sample sizes varied from 3.6 to 7.2 mm for the lattice region, and additional solid material was added to the top and bottom to make the total height 8 mm in all cases for simpler loading in the compression cell.

MicroCT scanning was done using laboratory nanoCT as described in Reference [21] using a Deben in-situ loading stage (CT500, Deben UK, London, UK) in a General Electric Nanotom scanner (Nanotom S, General Electric, Wunstorf, Germany). The sample sizes in this work were selected according to the maximum sample size of 10 mm and maximum loading force of 500 N of this loading stage. One sample design that did not fail up to 500 N was additionally subjected to compression tests on a different loading stage to obtain the yield strength. This was the smallest sample with the highest density (Figure 1, sample on the far left).

The microCT voxel size was selected as 4 µm, with 140 kV and 130 µA for the X-ray generation, using a 0.5 mm copper beam filtration and using continuous scanning mode, a total of 3600 images were recorded during a full rotation of the sample. Images were further analyzed in Volume Graphics VGSTUDIO MAX 3.2 (version 3.2, Volume Graphics, Heidelberg, Germany) [22]. Wall thickness analysis used in this work was done with the sphere-method. Due to file sizes and limited computing power, the combined images were resampled in VGSTUDIO MAX to a 10 µm voxel size and 8 bit data depth to reduce file sizes and memory usage to ease the image analysis.

## 3. Results and Discussion

**Figure 2:**
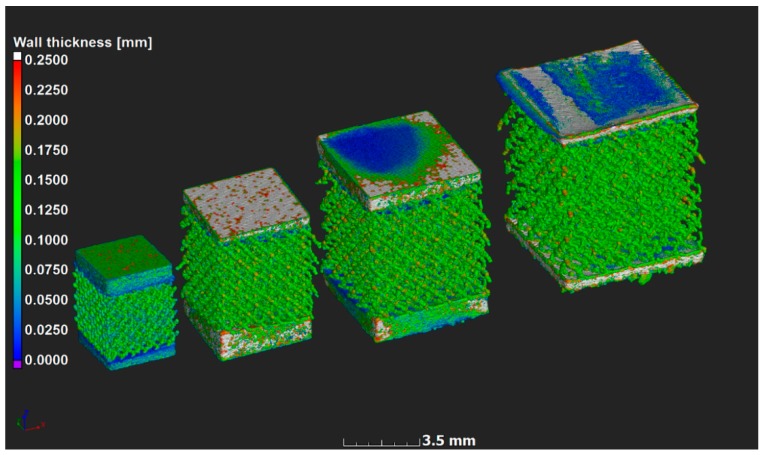
MicroCT scan data 3-D rendering with strut thickness color coding on the microlattices. Models had a 0.6, 0.8, 1.0 and 1.2 mm unit cell size from left to right respectively; here the top and bottom of the samples were slightly cropped as the lattice area was scanned only. Dark blue indicates thin local walls and red indicates thick walls, thickest parts were excluded to highlight the important aspect. All lattice struts have similar thickness (0.1–0.15 mm).

Samples were manufactured successfully, but microCT analyses showed that the strut thickness across the models did not vary as expected; this is shown in Figure 2 using a strut thickness analysis, analogous to Figure 1. Irregularities were expected at this scale due to various practical limitations that exist when producing small intricate parts during LPBF. The cause of such irregularities can be explained when looking at the minimum size of the designed features with regard to the combined effects of the laser spot size, building direction, layer thickness, and the implemented scanning strategy for core, overhangs, and top surfaces, all having an effect on the amount of detail that could be obtained. Small features were also governed by the single track’s width and attached powder particles, which in turn were limited by powder particle size distribution. Accuracy of small overhangs was not only dependent on the layer thickness but also on the loose powder and the inability of the molten pool to penetrate into solid material of the lattice. Therefore, irregular surfaces below the struts were expected [23].

**Figure 3:**
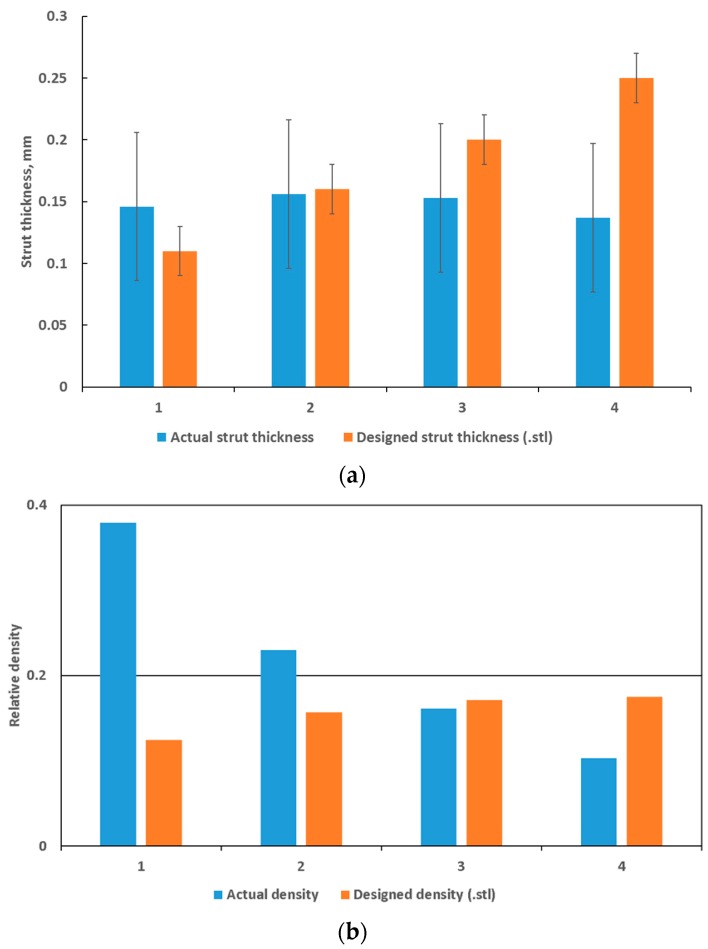
Dimensional assessment of (**a**) actual versus designed strut thickness and (**b**) actual versus designed total density compared to the designed values. Maximum and minimum values are shown as an error margin but indicates the variability within a single strut, as measured manually from 3-D model .stl data and microCT data.

**Figure 4:**
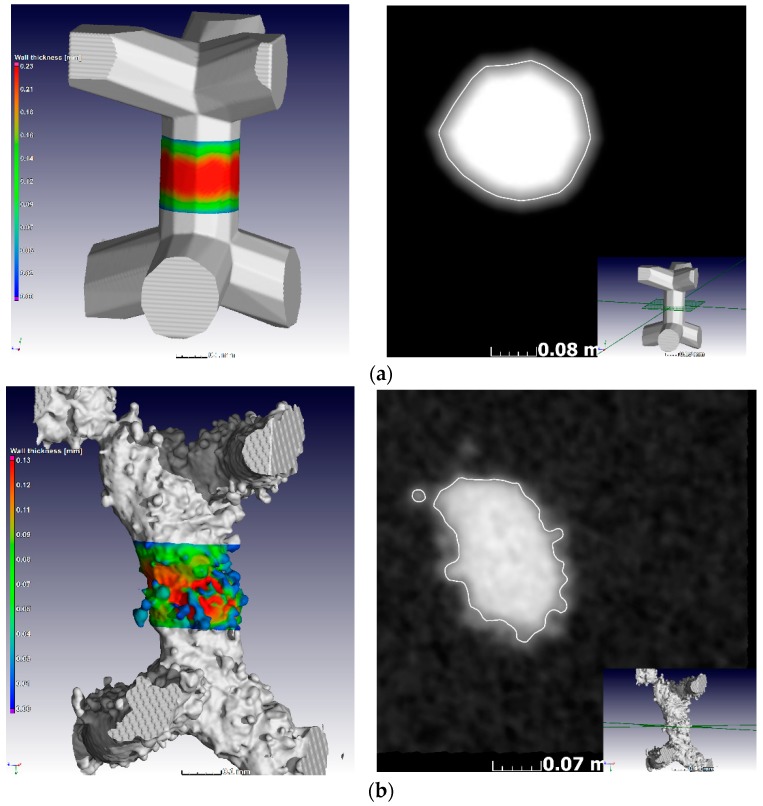
MicroCT voxel view of (**a**) designed and (**b**) manufactured struts and their cross-sections, indicating the actual morphology in each case.

In previous work [24], it was shown that at layer thickness 15–45 µm and similar Ti6Al4V powder and process-parameters, the width of the track was 100–150 µm. The small size of the designed struts, which are close to the single-track width of the laser melting track width, combined with the scanning strategy of the LPBF system used apparently did not allow for variations and these lattices were seemingly all produced with a similar strut thickness deviating from the design thickness as shown in Figure 3a and Figure 4. On the one hand, .stl triangulation of small structures led to an irregular shape of the struts (Figure 4a). Second, analysis of the scanning strategy showed that designed fine structures (less than 300 µm) were scanned by the laser as single lines with process parameters for the skin (contouring). Thus, struts in each of the produced sets of 0.6–1.2 mm units were similar and had thickness of 90–220 µm and they were very rough (Figure 4b). For the total density, the result was that the larger lattice had larger pore spaces, making its actual density lower than designed, as shown in Figure 3b.

**Figure 5:**
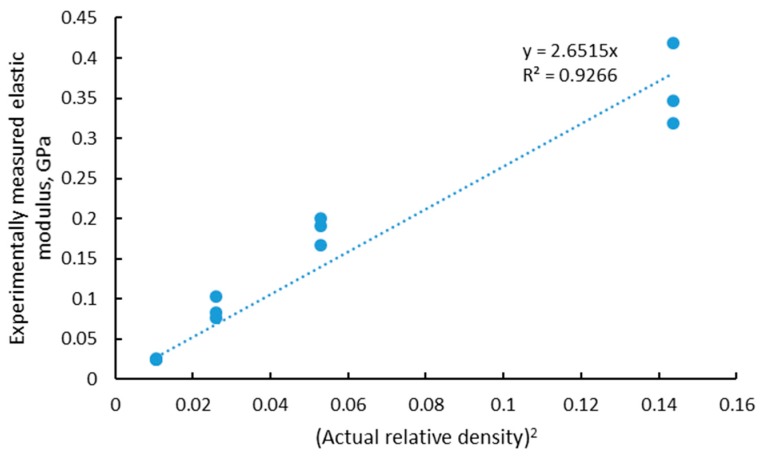
Relationship for experimentally measured elastic modulus and squared relative density of the manufactured LPBF lattices.

As explained in the previous section, the simplified model of Ashby–Gibson for open cell foams indicates a linear correlation between the elastic modulus and the square of the density. This was found experimentally in this case with a slope of approximately 2.8, as shown in Figure 5.

The value of 2.8 for the slope was within the expected range of 0.1–4. Previous work with larger lattices built using the same material process parameters showed that the experimental elastic modulus values were 10 and 20 GPa for 50% density lattice structures of two designs, diagonal and rhombic, respectively [17]. This relates to values for alpha (the slope) of 0.35 to 0.7. We can therefore speculate that as the strut thickness reduces, the effect of the rough and irregular surface plays an increasingly important role, increasing the slope and making the structure’s mechanical properties more sensitive to changes in density. What is interesting to note here is that since the lattice properties follow the density, the smallest lattice of 3.6 mm (unit cell of 0.6 mm) was the strongest; the yield strength is shown in Table 1 together with the actual relative porosity as measured by microCT. This was due to the similar strut thickness of the four models but shorter strut lengths and hence higher density for the smallest model. This also shows that at this scale, the strength and elastic modulus is strongly correlated with the actual porosity (or density).

**Figure 6:**
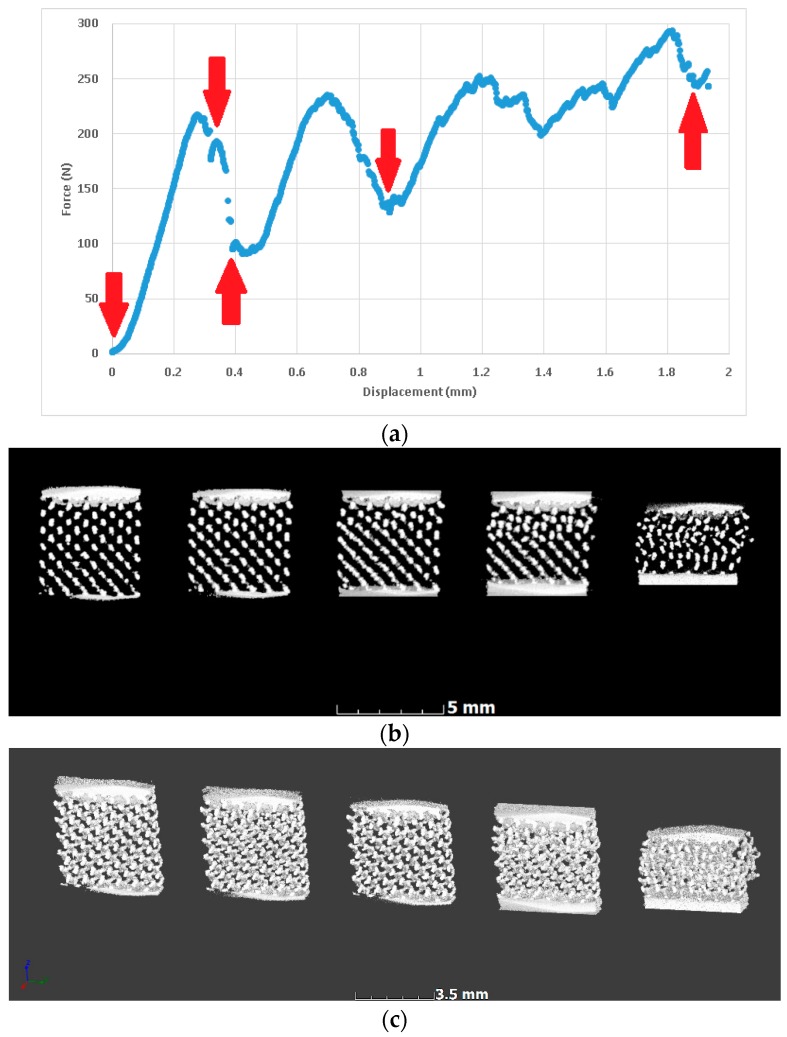
In situ deformation imaging of a lattice (0.8 mm unit cell) at the selected points during yielding; steps are shown as arrows in the force-displacement curve. (**a**) Force-displacement with red arrows indicating steps for microCT scans (stop); (**b**) MicroCT slice images at each step showing collapse; (**c**) The corresponding microCT 3-D images of the lattice at each step (cropped).

In situ compression allowed imaging of the same lattice prior to full densification, first before loading, then directly after initial yielding, and at a few more representative steps during yielding. This is shown in Figure 6, where red arrows indicate the positions where the loading was stopped and microCT scans were performed. The resulting microCT data is represented for the aligned volumes, with side-by-side slice images through the middle of the lattice, and with 3-D views of the entire lattice. These images indicate that yielding occurred gradually and progressively as struts collapsed in this type of lattice.

**Figure 7:**
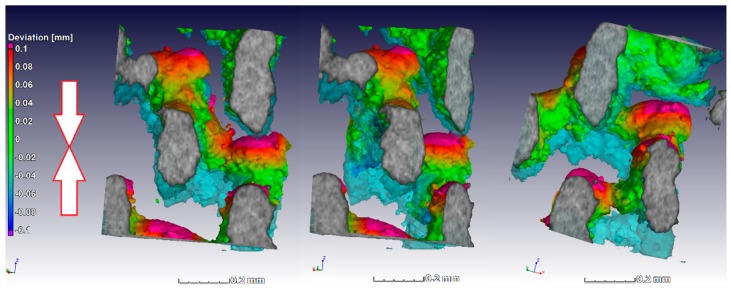
Three different angular views of the same internal location showing yielding behavior of individual struts and color coding indicating the largest deformation relative to the unloaded state (deformation upwards in image, where the unloaded state is semi-transparent blue). Loading direction is indicated by arrows.

The alignment of the scans was simplified by the fact that the sample stayed in the same location in the scan system, and as the load cell works by moving the bottom upwards, the deformation could be imaged more closely on individual struts by making the unloaded scan transparent and visualizing the loaded image. This is done in Figure 7 for a small section (approx. 0.5 mm) to visualize the deformation and collapse of individual struts, in this case taken from the middle (away from the edge of the lattice) near the top of the sample where collapse first occurred. The light blue transparent struts shown in Figure 7 show the unloaded sample, while the third scan in the series is shown here in solid rendering (this is at the first yield dip, the third arrow in Figure 6). The color coding applied to the loaded sample image is a nominal–actual comparison; this quantitatively shows the deformation value (in red was where largest deformation was relative to the unloaded sample). Besides collapse, the largest deformations occurred at the strut junctions. Similar results were reported for Ti6Al4V lattices in Reference [25] where digital image correlation (of external surface of lattice) was used during compression testing of diamond-type lattices. It was found that fracture occurs exclusively at the nodes in this design, and similar layered collapse was observed. The three images are the same region from different viewing angles.

## 4. Conclusions

This paper reported the mechanical properties of a series of microlattices with struts near the single-track width of the laser powder bed fusion system used to produce them. The results show that such lattices could be produced successfully but the small strut thickness deviated from the designed value. It was shown that, in the limited range investigated here, the mechanical properties of microlattices produced by LBPF were strongly dependent on actual density and could therefore be predicted with some confidence using this measure alone. Compared to larger lattices, the dependence of the mechanical properties was stronger with density (higher slope in the Ashby–Gibson equation). In situ microCT imaging demonstrated that the largest deformations under compression occurred at the strut junctions. These images represent the first in situ images of a full microlattice structure’s yielding behavior.

## Figures and Tables

**Table 1:** Experimental data.

Unit Cell Design (mm)	Actual Relative Porosity (%)	Compressive Elastic Modulus (MPa)	Compressive Strength (MPa)	Maximum Load (N)
0.6	63	346 418 318	51.1 54.8 53.4	662 710 692
0.8	77	190 167 200	9.1 9.9 10.1	209 227 232
1.0	84	83 77 102	3.3 3.9 3.6	117 139 130
1.2	90	26 25 24	1.0 0.9 1.1	53 46 56

## References

[1] Schmidt M., Merklein M., Bourell D., Dimitrov D., Hausotte T., Wegener K., Overmeyer L., Vollertsen F., Levy G.N. (2017). Laser based additive manufacturing in industry and academia. CIRP Ann..

[2] DebRoy T., Wei H.L., Zuback J.S., Mukherjee T., Elmer J.W., Milewski J.O., Beese A.M., Wilson-Heid A., De A., Zhang W. (2017). Additive manufacturing of metallic components—Process, structure and properties. Prog. Mater. Sci..

[3] Yadroitsev I., Gusarov A., Yadroitsava I., Smurov I. (2010). Single track formation in selective laser melting of metal powders. J. Mater. Process. Technol..

[4] Du Plessis A., Yadroitsev I., Yadroitsava I., Le Roux S. (2018). X-ray micro computed tomography in additive manufacturing: A review of the current technology and applications. 3D Print. Addit. Manuf.

[5] Yadroitsev I., Krakhmalev P., Yadroitsava I., Du Plessis A. (2018). Qualification of Ti6Al4V ELI Alloy Produced by Laser Powder Bed Fusion for Biomedical Applications. JOM.

[6] Tan X.P., Tan Y.J., Chow C.S.L., Tor S.B., Yeong W.Y. (2017). Metallic powder-bed based 3D printing of cellular scaffolds for orthopaedic implants: A state-of-the-art review on manufacturing, topological design, mechanical properties and biocompatibility. Mater. Sci. Eng. C.

[7] Zhang X.-Y., Fang G., Zhou J. (2017). Additively Manufactured Scaffolds for Bone Tissue Engineering and the Prediction of their Mechanical Behavior: A Review. Materials.

[8] Dong G., Tang Y., Zhao Y.F. (2017). A Survey of Modeling of Lattice Structures Fabricated by Additive Manufacturing. J. Mech. Des..

[9] Gibson L., Ashby M. (1999). Cellular Solids: Structure and Properties.

[10] Ashby M., Evans T., Fleck N., Hutchinson J. (2000). Metal Foams: A Design Guide.

[11] Parthasarathy J., Starly B., Raman S., Christensen A. (2010). Mechanical evaluation of porous titanium (Ti6Al4V) structures with electron beam melting (EBM). J. Mech. Behav. Biomed. Mater..

[12] Sallica-Leva E., Jardini A.L., Fogagnolo J.B. (2013). Microstructure and mechanical behavior of porous Ti–6Al–4V parts obtained by selective laser melting. J. Mech. Behav. Biomed. Mater..

[13] Ahmadi S.M., Campoli G., Yavari S.A., Sajadi B., Wauthlé R., Schrooten J., Weinans H., Zadpoor A.A. (2014). Mechanical behavior of regular open-cell porous biomaterials made of diamond lattice unit cells. J. Mech. Behav. Biomed. Mater..

[14] Ahmadi S.M., Yavari S.A., Wauthle R., Pouran B., Schrooten J., Weinans H., Zadpoor A.A. (2015). Additively Manufactured Open-Cell Porous Biomaterials Made from Six Different Space-Filling Unit Cells: The Mechanical and Morphological Properties. Materials.

[15] Bobbert F.S., Lietaert K., Eftekhari A.A., Pouran B., Ahmadi S.M., Weinans H., Zadpoor A.A. (2017). Additively manufactured metallic porous biomaterials based on minimal surfaces: A unique combination of topological, mechanical, and mass transport properties. Acta Biomater..

[16] Du Plessis A., Yadroitsava I., Yadroitsev I., le Roux S., Blaine D. (2018). Numerical comparison of lattice unit cell designs for medical implants by additive manufacturing. Virtual Phys. Prototyp..

[17] Du Plessis A., Yadroitsava I., Yadroitsev I. (2018). Ti6Al4V lightweight lattice structures manufactured by laser powder bed fusion for load-bearing applications. Opt. Laser Technol..

[18] Carlton H.D., Lind J., Messner M.C., Volkoff-Shoemaker N.A., Barnard H.S., Barton N.R., Kumar M. (2017). Mapping local deformation behavior in single cell metal lattice structures. Acta Mater..

[19] Qiu C., Yue S., Adkins N.J., Ward M., Hassanin H., Lee P.D., Withers PJAttallah M.M. (2015). Influence of processing conditions on strut structure and compressive properties of cellular lattice structures fabricated by selective laser melting. Mater. Sci. Eng. A.

[20] Materialise. https://www.materialise.com/en/software/magics.

[21] Du Plessis A., le Roux S.G., Guelpa A. (2016). The CT Scanner Facility at Stellenbosch University: An open access X-ray computed tomography laboratory. Nucl. Instrum. Methods Phys. Res. Sect. B Beam Interact. Mater. Atoms..

[22] Volume graphics. https://www.volumegraphics.com/en/products/vgstudio-max.html.

[23] Kouprianoff D., du Plessis A., Yadroitsava I., Yadroitsev I. Destructive and nondestructive testing on small and intricate SLM components. Proceedings of the 18th Annual International RAPDASA Conference.

[24] Yadroitsava I., Els J., Booysen G., Yadroitsev I. (2015). Peculiarities of single track formation from TI6AL4V alloy at different laser power densities by SLM. S. Afr. J. Ind. Eng..

[25] Liu F., Zhang D.Z., Zhang P., Zhao M., Jafar S. (2018). Mechanical Properties of Optimized Diamond Lattice Structure for Bone Scaffolds Fabricated via Selective Laser Melting. Materials.

